# Co-expression network analysis and cis-regulatory element enrichment determine putative functions and regulatory mechanisms of grapevine ATL E3 ubiquitin ligases

**DOI:** 10.1038/s41598-018-21377-y

**Published:** 2018-02-16

**Authors:** Darren C. J. Wong, Pietro Ariani, Simone Castellarin, Annalisa Polverari, Elodie Vandelle

**Affiliations:** 1 Wine Research Centre, University of British Columbia, 2205 East Mall, Vancouver, BC V6T 1Z4 Canada; 2 Dipartimento di Biotecnologie, Università degli Studi di Verona, Verona, 37134 Italy; 3 Present Address: Ecology and Evolution, Research School of Biology, The Australian National University, Acton, ACT 2601 Australia

## Abstract

*Arabidopsis thaliana Toxicos en Levadura* (ATL) proteins are a subclass of the RING-H2 zinc finger binding E3 ubiquitin ligases. The grapevine (*Vitis vinifera*) ATL family was recently characterized, revealing 96 members that are likely to be involved in several physiological processes through protein ubiquitination. However, the final targets and biological functions of most ATL E3 ligases are still unknown. We analyzed the co-expression networks among grapevine ATL genes across a set of transcriptomic data related to defense and abiotic stress, combined with a condition-independent dataset. This revealed strong correlations between ATL proteins and diverse signal transduction components and transcriptional regulators, in particular those involved in immunity. An enrichment analysis of *cis*-regulatory elements in ATL gene promoters and related co-expressed genes highlighted the importance of hormones in the regulation of ATL gene expression. Our work identified several ATL proteins as candidates for further studies aiming to decipher specific grapevine resistance mechanisms activated in response to pathogens.

## Introduction

The ubiquitin proteasome system (UPS) pathway, a major regulatory network to integrate internal and external signals in plants^1^, is highly conserved among eukaryotes, and is based on an integrated three-step conjugation cascade: E1 > E2 > E3^2^. The outcome of this enzyme cascade is the covalent binding of one or more ubiquitin molecules to a substrate protein, resulting in mono-ubiquitinylation or poly-ubiquitinylation, respectively. In this process, the ubiquitin ligase E3 is responsible for substrate recognition and facilitates the transfer of ubiquitin from E2 to the substrate protein^3^. *Arabidopsis thaliana* genome contains more than 1400 putative ubiquitin ligase E3 genes^4^ and this large number of possible ligases allows many potential combinations of enzymes in the cascade, which confers extreme flexibility and thus helps the plant to respond precisely to environmental changes and stresses. The E3 ubiquitin ligases can be assigned to various groups based on the presence of specific domains. The three most important classes are the HECT, U-box and RING families^3^.

Among the family of RING (Really Interesting New Gene) E3 ligases, a particular sub-family, named *Arabidopsis Toxicos en Levadura* (ATL)^5^ and characterized by a specific variant of the RING domain known as RING-H2^6^, has been shown to be important for plant growth as well as for biotic or abiotic stress responses^7,8^. For instance, AtATL2, the first member identified in *A*. *thaliana*, and NtACRE132, its orthologue in tobacco, as well as AtATL6, AtATL9 and AtATL31, are rapidly induced at the transcriptional level by elicitors such as chitin and cellulases^2,9–11^ and the overexpression of AtATL2 or its orthologue in poplar PtaRHE1 up-regulates defense-related gene expression^9,12^. In the same manner, AtATL55/RING1 is induced following exposure to *Pseudomonas syringae* DC3000 AvrRpm1, the fungal toxin fumonisin B1 and chitin^10,13^. The tomato LeATL6 and pepper CaRING1 proteins also play a role in pathogen resistance via crosstalk between the SA and ethylene/JA pathways, and are necessary for the hypersensitive response^14,15^. Finally, the potato StRFP1 protein contributes to broad-spectrum resistance against *Phytophthora infestans*^16^.

In contrast, AtATL78 and AtATL80 are negative regulators of the cold stress response^17,18^. Other ATL proteins facilitate more diverse cellular processes, such as the regulation of programmed cell death during root development (EL5), the intraplastidial trafficking at thylakoid membranes (AtATL25/NIP2) and the repression of the photoperiod response (AtATL62)^9^. Finally, the function of a given ATL protein is not necessarily restricted to a single process. Accordingly, AtATL6 and AtATL31 not only respond to elicitors but also to carbon/nitrogen status^19^, and similarly AtATL9 responds to elicitors but also mediates the endoplasmic reticulum-associated degradation (ERAD) of misfolded proteins^11^.

The ATL family has been fully characterized in *A*. *thaliana* and rice^6^ and was very recently identified in grapevine (*Vitis vinifera*), revealing 96 putative ATL genes^20^. At the functional level, only the EIRP1 protein from the wild species *Vitis pseudoreticulata* has been described thus far, in a report demonstrating its interaction with the defense-related transcription factor VpWRKY11^21^. Furthermore, transcriptomic analysis has shown that eight putative ATL genes are upregulated in response to infection with *Plasmopara viticola* specifically in the resistant species *Vitis riparia*, suggesting a role in the defense response for these ATL proteins also in grapevine^22^.

The analysis of co-expression networks can help to build biological hypotheses from large amounts of grapevine transcriptomic data^23–26^. Given the scattered information concerning the roles of individual ATL genes in different species, we sought to determine the functions and relationships of the grapevine ATL family on a broader scale by characterizing the co-expression networks of all 96 grapevine ATL genes. Gene regulation in response to physiological and developmental changes can be inferred from correlations among different co-expression networks. Grapevine ATL genes are expressed under diverse physiological conditions^20^, so we used multiple datasets representing abiotic and biotic stress as well as a condition-independent dataset. To unravel the regulatory mechanisms controlling ATL community clusters, we also identified *cis*-regulatory elements that are enriched in the promoters of the entire ATL gene family and in specific network modules. These data provide a basis for the empirical functional analysis of ATL genes that may promote disease resistance in grapevine, which ultimately could lead to the development of hardier varieties.

## Results

### Overview of ATL condition-independent and condition-dependent co-expression networks

To gain insight into the potential coordinated and regulated molecular pathways of diverse grapevine ATL proteins, a large gene expression compendium (DatasetCI) encompassing a wide range of tissues (e.g. berries, flowers, leaves and roots), relevant developmental stages, and types of stress (e.g. drought and pathogens) was constructed using publicly available RNA-Seq data (see Supplementary Material). In addition, two contrast matrices representing relevant abiotic (DatasetA) and biotic (DatasetB) stress conditions were constructed. The abiotic dataset compared grapevine plants exposed to water deficit^27–29^, heat stress/acclimation^30^, shading, and gibberellic acid treatment at bloom in flowers/inflorescences^31^, whereas the biotic dataset included grapevine plants infected with *Botrytis cinerea*^32^, *Erysiphe necator*^33^ or *P*. *viticola*^34^, or infested by the spider mite *Tetranyhus urticae*^35^.

Based on the Gini correlation coefficient (GCC) values for each ATL gene pair in each dataset, the top 100 ranked genes (descending GCC values) were selected for further analysis (Supplementary Table S1). To validate the selection of only the top 100 ranked co-expressed genes, we also determined the statistical significance of GCC values and estimated the incidence of false positive correlations (Supplementary Table S2). In the condition-independent dataset, 69.2% (6,709 pairs) and 89.6% (8,690 pairs) of all GCC values within the top 100 co-expressed ATL genes showed significant correlations at the 5% and 10% false positive rates, respectively. Nearly all the gene pairs in both the abiotic (99.9%, 9,384 pairs) and biotic (98.9%, 9,292 pairs) datasets showed significant correlations at the 5% false positive rate.

### Distribution, frequency and enrichment of functional categories in the ATL subnetworks of the condition-independent, biotic and abiotic datasets

The distribution and frequency of MapMan BINs^36^ associated with the 96 ATL subnetworks revealed the predominant representation of certain categories, including BIN 26 (miscellaneous enzyme families), BIN 27 (regulation of transcription), BIN 29 (protein metabolism) and BIN 30 (signaling), and to a lesser extent BIN 10 (cell wall), BIN 11 (lipid metabolism), BIN 16 (secondary metabolism), BIN 17 (hormones), BIN 31 (cell) and BIN 34 (transport) (Supplementary Figs S1 and S2). These results showed that most of the ATL subnetworks share common BIN enrichments regardless of the physiological conditions, suggesting conserved functions and/or regulation among ATL family members and thus an important correlation between ATL functions and particular cellular processes.

**Figure 1:**
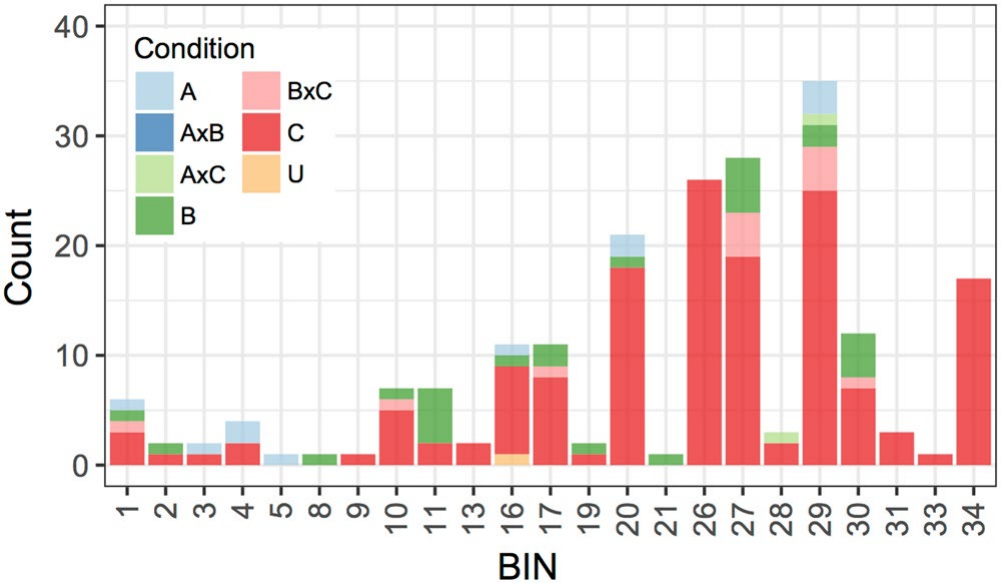
Number of grapevine ATL-centered co-expression clusters enriched in MapMan BIN categories under different conditions. MapMan BIN categories were considered significantly enriched within the corresponding ATL subnetworks with a false discovery rate (FDR) threshold of <0.05. Each ATL subnetwork is represented only once in a Venn format to remove redundancy. A, abiotic; B, biotic; C, condition-independent; U, common to all; AxB, common to abiotic and biotic; AxC, common to abiotic and condition-independent; BxC, common to biotic and condition-independent.

More precisely, the number of subnetworks enriched for a given BIN term and the median number of genes significantly associated with a certain BIN term across ATL subnetworks may indicate the predominant functions associated with the ATL family. The analysis of BIN enrichment in ATL subnetworks in each dataset (condition-independent, abiotic and biotic) was visualized using a Venn format to avoid redundancy, taking into account the fact that one subnetwork could be enriched for a given BIN in more than one dataset (Fig. 1 and Supplementary Table S3). Collectively, 70, 22 and 38 ATL subnetworks were significantly enriched (false discovery rate (FDR) < 0.05) for at least one MapMan BIN in the condition-independent, abiotic and biotic datasets, respectively, representing a total of 82 ATL subnetworks. Moreover, BIN 26 (miscellaneous enzyme families) and BIN 34 (transport) were enriched only in the condition-independent dataset, in 26 and 17 ATL subnetworks, respectively. Similarly, BIN 31 (cell), BIN 13 (amino acid metabolism), BIN 33 (development) and BIN 9 (mitochondrial electron transport/ATP synthesis) were enriched solely in the condition-independent dataset, but only in 1–3 ATL subnetworks. In contrast, only BIN 5 (fermentation) was enriched solely under abiotic stress conditions, whereas BIN 8 (TCA/organic acid transformation) and BIN 21 (redox) were enriched specifically under biotic stress, each of them in only one ATL subnetwork. This indicates that specific features for some ATL proteins could also be observed under particular conditions. Moreover, although BIN 29 (protein metabolism) was enriched in a total of 35 ATL subnetworks, none were common to all conditions. This indicates that, in accordance with the proposed sub-functionalization of ATL genes^20^, a given function may be carried out by different ATL genes depending on the conditions.

The specificity of ATL function under particular conditions is highlighted by looking at common ATL subnetworks showing BIN enrichment across different datasets. Indeed, only one subnetwork (*VviATL149*, VIT_12s0028g02530) was common among all datasets and was enriched in genes related to secondary metabolism (BIN 16), with six genes encoding stilbene synthases, two genes encoding resveratrol synthases, and five genes encoding laccases. Moreover, two ATL subnetworks (*VviATL63*, VIT_06s0004g06930 and *VviATL119*, VIT_08s0040g00310) were similarly enriched for BIN 28 (DNA processing) and BIN 29 (protein metabolism) in both the abiotic and condition-independent datasets, whereas 10 ATL subnetworks (*VviATL100*, VIT_08s0040g02950; *VviATL163*, VIT_05s0049g00480; *VviATL156*, VIT_05s0077g01970; *VviATL3*, VIT_09s0002g00220; *VviATL149*, VIT_12s0028g02530; *VviATL97*, VIT_11s0016g03190; *VviATL114*, VIT_04s0008g04280; *VviATL115*, VIT_09s0002g05120; *VviATL139*, VIT_18s0122g00870; and *VviATL84*, VIT_06s0004g00120) were similarly enriched for the same BINs (1, 10, 17, 30, 27 and 29) in both the biotic and condition-independent datasets. Interestingly, no subnetwork was shared between the abiotic and biotic datasets, indicating that ATL proteins display a certain specificity towards particular stress responses.

The subnetworks of a given ATL gene can also be enriched for different BINs depending on the conditions. For example, the *VviATL23a* (VIT_18s0001g01060) subnetwork was enriched for BIN 5 (fermentation) and BIN 4 (glycolysis) in the abiotic dataset, but for BIN 9 (mitochondrial electron transport) in the condition-independent dataset. BIN 30 (signaling) was enriched only in the condition-independent and biotic datasets, although many subnetworks identified in the abiotic dataset include genes related to this functional category with a lower frequency (Supplementary Fig. S2).

**Figure 2:**
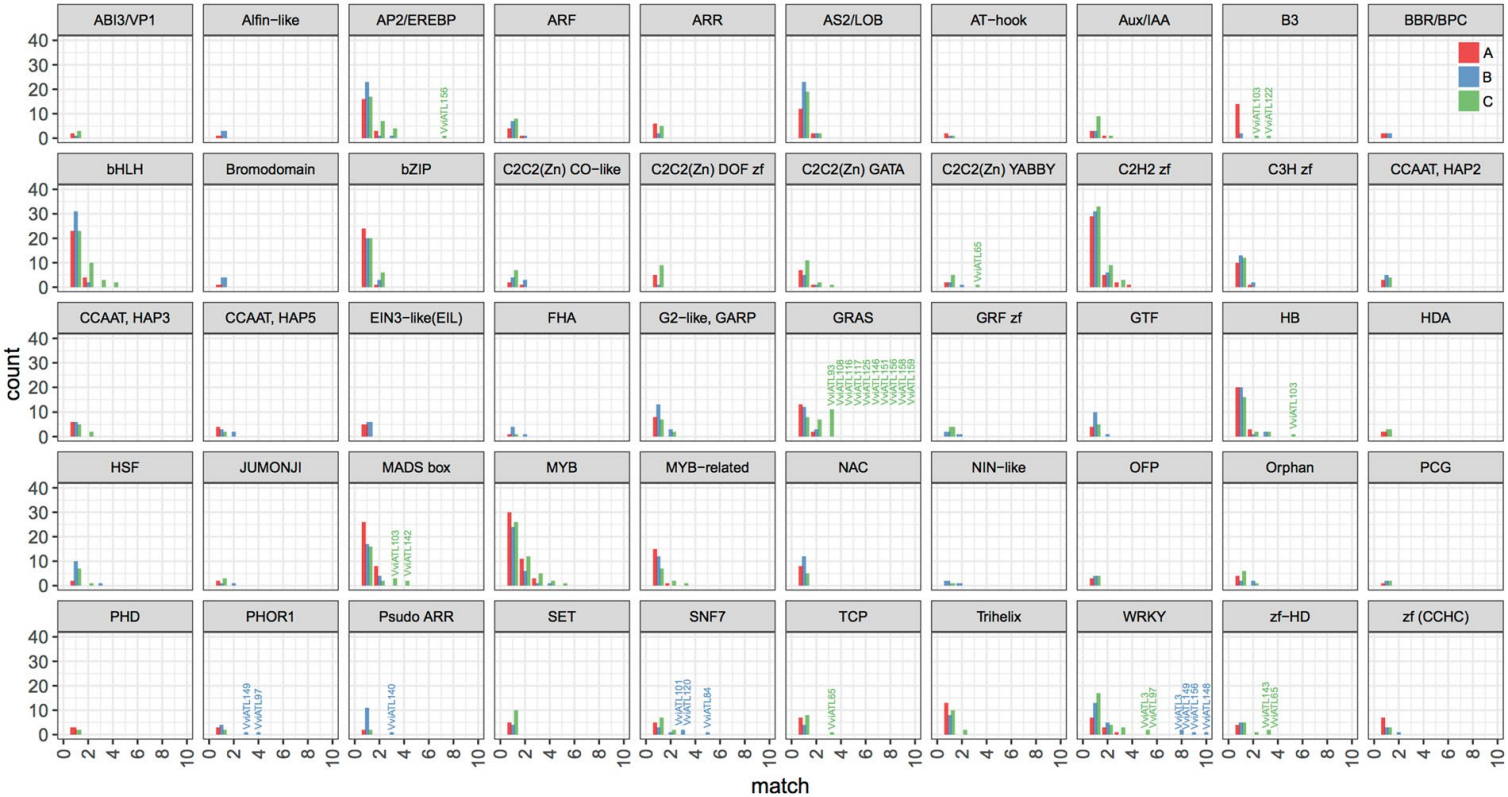
Histogram summarizing transcription factor frequencies in grapevine ATL subnetworks in the three datasets. The x-axis (match) represents the number of transcription factors representing a given family co-expressed with each ATL. The y-axis (count) represents the number of ATL subnetworks displaying a given number of co-expressed transcription factors. ATL subnetworks in which particular transcription factor families are enriched (FDR < 0.05) are indicated in the histogram.

The co-expression of ATL genes with transcription factors (BIN 27.3) deserves particular attention. Figure 2 shows the number of genes in each transcription factor family co-regulated with the ATL family overall across the different datasets. More than 30 ATL subnetworks include 1–5 co-regulated genes from each of the following transcription factor families: MYB (BIN 27.3.25), C2H2 zinc fingers (27.3.11), and bHLH (27.3.6). Up to 10 genes representing the WRKY (BIN 27.3.32) and AP2/ERF (BIN 27.3.3) families were co-regulated in more than 20 subnetworks. Significant enrichment was observed for the WRKY transcription factor family in two subnetworks of the condition-independent dataset (*VviATL3* and *VviATL97*) and four subnetworks of the biotic dataset (*VviATL3*, *VviATL148*, *VviATL149*, and *VviATL156*). Similarly, the AP2/ERF transcription factor family was enriched in one condition-independent subnetwork (*VviATL156*) (Supplementary Table S4). However, no enrichment was observed in the abiotic dataset due to the poor representation of WRKY transcription factors among co-expressed genes (11 genes in 11 ATL subnetworks) compared to the condition-independent and biotic datasets. Although not significantly enriched in any subnetwork, MYB was the most abundantly represented transcription factor family under all conditions, with 39–44 MYB genes co-regulated with 39–49 ATL genes, depending on the dataset (Supplementary Table S1). In contrast, although GRAS transcription factors were represented less frequently than other families, they were significantly enriched in as many as 10 ATL subnetworks (*VviATL93*, *VviATL108*, *VviATL116*, *VviATL117*, *VviATL125*, *VviATL146*, *VviATL151*, *VviATL156*, *VviATL158*, and *VviATL159*) in the condition-independent dataset, particularly GRAS8a, GRASV2a, GRASV1c, GRASV1d, LISCL3, LISCL6, PAT3, PAT4, PAT6, PAT8 and SCR3 (based on the most recent GRAS family nomenclature in grapevine)^37^. Other transcription factor families were enriched in 1–3 subnetworks of the condition-independent and biotic datasets: B3 (*VviATL103* and *VviATL122*), C2C2(Zn) YABBY (*VviATL165*), HB (*VviATL103*), MADS box (*VviATL103* and *VviATL142*), PHOR1 (*VviATL97* and *VviATL149*), pseudo ARR (*VviATL140*), SNF7 (*VviATL84*, *VviATL101* and *VviATL120*) and zf-HD (*VviATL65* and *VviATL143*) (Fig. 2).

### Enrichment of cis-acting regulatory elements in ATL gene promoters

**Figure 3:**
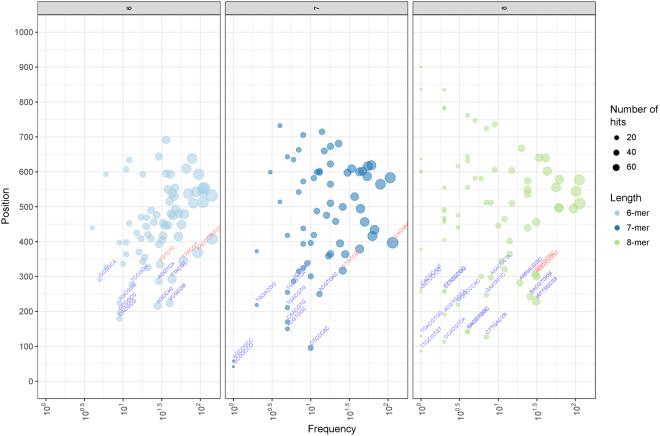
Frequency of *cis*-acting regulatory elements (CAREs) in grapevine ATL promoters. The y-axis represents the median position of each CARE from the transcriptional start site in the ATL promoter and the x-axis represents the total frequency of each CARE in all grapevine ATL promoters. Ball color represents the length of the motifs (green, 6-mers; purple, 7-mers; orange, 8-mers), whereas ball size indicates the number of non-redundant grapevine ATL promoters containing a given motif.

To investigate the influence of specific promoter motifs, and thus specific transcription factor families, in the regulation of ATL genes, the promoter regions of all grapevine ATL genes were analyzed to identify common *cis*-acting regulatory elements (CAREs) (Fig. 3 and Supplementary Table S5). We used the positional bias score (Z-score)^38^, which takes into account the length of CAREs, their frequency and their position within the upstream promoter. This allowed the ranking of CAREs according to their Z-scores, indicating their potential biological importance among the 96 grapevine ATL genes as a whole. The top 20 ranking CAREs included one calmodulin-binding motif (VCGGCB, CGCGBOXAT), three NAC sites (TTACGT, ANAC_C1b; TTRCGT, ANAC_C1con; TTCCTT, ANAC_C3b), two bZIP motifs (BACGTGKM, ABRE-like binding site motif; ACGTGKC, ACGTABREMOTIFA2OSEM), one MYB motif (GNATATNC, P1BS), one bHLH motif (CACGCG, MYC2–5), one AP2/ERF motif (GCCGCC, GCCCORE), one E2F motif (WTTSSCSS, E2FCONSENSUS) and one WRKY motif (CTTGACYR, WRKY18). The top-ranking CARE was the CGCGBOXAT motif, found in 26 ATL promoters and known as a Ca^2+^/calmodulin regulatory element. Ten other motifs enriched among the ATL promoters (SORLIP1, ANAC_C1b, ABRE-like binding site motif, ANAC_C1con, ANAC_C3b, WRKY18, MYC2–5, GCCCORE, ARFAT and ACGTABREMOTIFA2OSEM) are involved in hormone responses, with eight involved in abscisic acid (ABA) regulation and the other two mediating responses to JA and ethylene. Extending the analysis to another 25 well-described motifs with lower but still significant Z-scores revealed further ATL regulation by hormones (particularly ABA) as well as light-dependent and stress-dependent CAREs, CAREs related to growth and development (LBD16, UP2ATMSD, KAN4, ANAC46) and CAREs mediating tissue-specific expression (BS1EGCCR, BP5OSWX, GCN4OSGLUB1). Interestingly, motifs related to growth and development were found in only a few ATL genes, suggesting that a small number of ATLs have specific roles in these processes.

### Enrichment of CAREs in the abiotic, biotic and condition-independent ATL datasets

**Figure 4:**
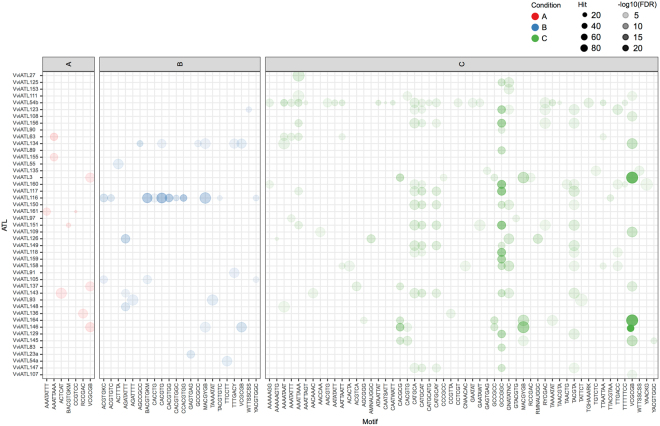
Enrichment of *cis*-acting regulatory elements (CAREs) in grapevine ATL subnetworks under different conditions. The different ATL subnetworks displaying CARE enrichment (FDR < 0.01) are indicated on the left. A, B and C as well as ball color indicate the abiotic (red), biotic (blue) and condition-independent (green) datasets, respectively. Ball size represents the number of genes in each ATL subnetwork containing a given CARE (20–80), whereas ball opacity represents the false discovery rate (FDR) expressed as −log10(FDR), thus the higher values (represented by darker shading) indicate a higher statistical significance.

CARE enrichment analysis was then carried out in the various ATL subnetworks of the three different datasets to identify promoter elements correlated with specific functions (Fig. 4 and Supplementary Table S6). In total, 45 ATL subnetworks displayed significant enrichment for at least one CARE. In the abiotic dataset, only nine ATL subnetworks showed significant CARE enrichment. Subnetwork *VviATL151* (VIT_11s0016g03420) was enriched for the BACGTGKM motif (an ABA-responsive element) whereas subnetwork *VviATL155* (VIT_07s0005g00710) was enriched for the PREATPRODH motif (response to hypo-osmolarity). Only one subnetwork (*VviATL126*, VIT_12s0028g01580) was enriched for two motifs: AHL12 (negative regulation of defense responses) and PALBOXAPC (response to elicitors and light). Finally, two motifs were found in more than one subnetwork, with Bellringer/Replumless/Pennywise.BS1.IN.AG enriched in two subnetworks (*VviATL136*, VIT_15s0024g01990 and *VviATL143*, VIT_13s0084g00140), and CGCGBOXAT enriched in three subnetworks (*VviATL91*, VIT_13s0019g01980; *VviATL55*, VIT_07s0191g00230 and *VviATL156*, VIT_05s0077g01970). In the biotic dataset, 13 ATL subnetworks showed significant CARE enrichment involving 22 motifs. Among these 13 networks, 10 displayed enrichment for one or two particular motifs, whereas *VvATL135* (VIT_08s0058g01270) was enriched primarily for CAREs representing ABA-related bZIP/BZR/BES transcription factors, *VviATL148* (VIT_14s0128g00120) was enriched primarily for CAREs representing AP2/bZIP/WRKY/CaM transcription factors, and *VviATL118* (VIT_12s0057g01330) was enriched for 11 CAREs, most related to ABA signaling (ACGTABREMOTIFA2OSEM, GADOWNAT, ABRE-like binding site motif, RAV1BAT, IRO2OS, ABREZMRAB28, EMBP1TAEM, ABREMOTIFAOSOSEM, ABRERATCAL and ABREATCONSENSUS) and the remaining motif (ACGTGMOTIF) was involved in light-dependent regulation and defense. These sites are broadly recognized by the bZIP, bHLH and BZR1 transcription factor families^39^. Among the enriched CAREs, four (NTBBF1ARROLB, SORLIP5AT, ANAC_C3b and E2FCONSENSUS) were unique to a given subnetwork, and two (RVE1–2 and LECPLEACS2) were only co-enriched in the *VviATL89* (VIT_06s0009g02350) subnetwork. Finally, in the condition-independent dataset, 38 ATL subnetworks were enriched for a total of 54 motifs. As observed in the other datasets, some ATL subnetworks were enriched for only one or two particular motifs whereas others featured more diversity. For example, 24 different motifs were statistically enriched in the *VviATL83* (VIT_17s0000g08400) subnetwork, corresponding mainly to binding sites for various transcriptional repressors (AHL12–1, AHL20–1, DOF5.7–1, KAN1) and hormone-responsive transcription factors (ATHB12, GARE2OSREP1, KAN1, LECPLEACS2, PYRIMIDINEBOXHVEPB1, RYREPEATBNNAPA, RYREPEATVFLEB4, T/GBOXATPIN2, WOX13–2). Some CAREs were found only in a few subnetworks, whereas others were shared among several subnetworks. For example, three motifs (one containing RYREPEATBNNAPA, RYREPEATGMGY2 and RYREPEATLEGUMINBOX elements; one for RAP2.6 binding, and the ACGTABOX element) were co-enriched in seven subnetworks (*VviATL146*, VIT_15s0046g00930; *VviATL159*, VIT_13s0019g00990; *VviATL149*, VIT_12s0028g02530; *VviATL151*, VIT_11s0016g03420; *VviATL150*, VIT_10s0003g00850; *VviATL155*, VIT_07s0005g00710; *VviATL125*, VIT_00s0349g00040), thus linking protein storage (development), hormone/stress responses and the sugar-dependent regulation of gene expression. Moreover, the RAP2.6 binding site was strongly enriched in 17 ATL subnetworks, suggesting that AP2/ERF transcription factors specifically binding to these sequences play an important role, as reported for *A*. *thaliana* RAP2.11 and RAP2.6^40^, and also other transcription factors that bind these sequences with lower affinity^39^. These AP2/ERF transcription factors may also play an important role in ABA/JA-dependent gene regulation and responses to saline and osmotic stress and pathogens, and may modulate the co-expressed ATL genes independently of the experimental conditions. Finally, the most significantly enriched motif was the CGCGBOXAT element involved in Ca^2+^/calmodulin regulation. This was enriched in the *VviATL156* subnetwork in all three datasets, suggesting a strong correlation between this network and Ca^2+^ signaling regardless of the conditions.

### Community clustering of co-expressed ATL genes in the condition-independent, biotic and abiotic datasets

**Figure 5:**
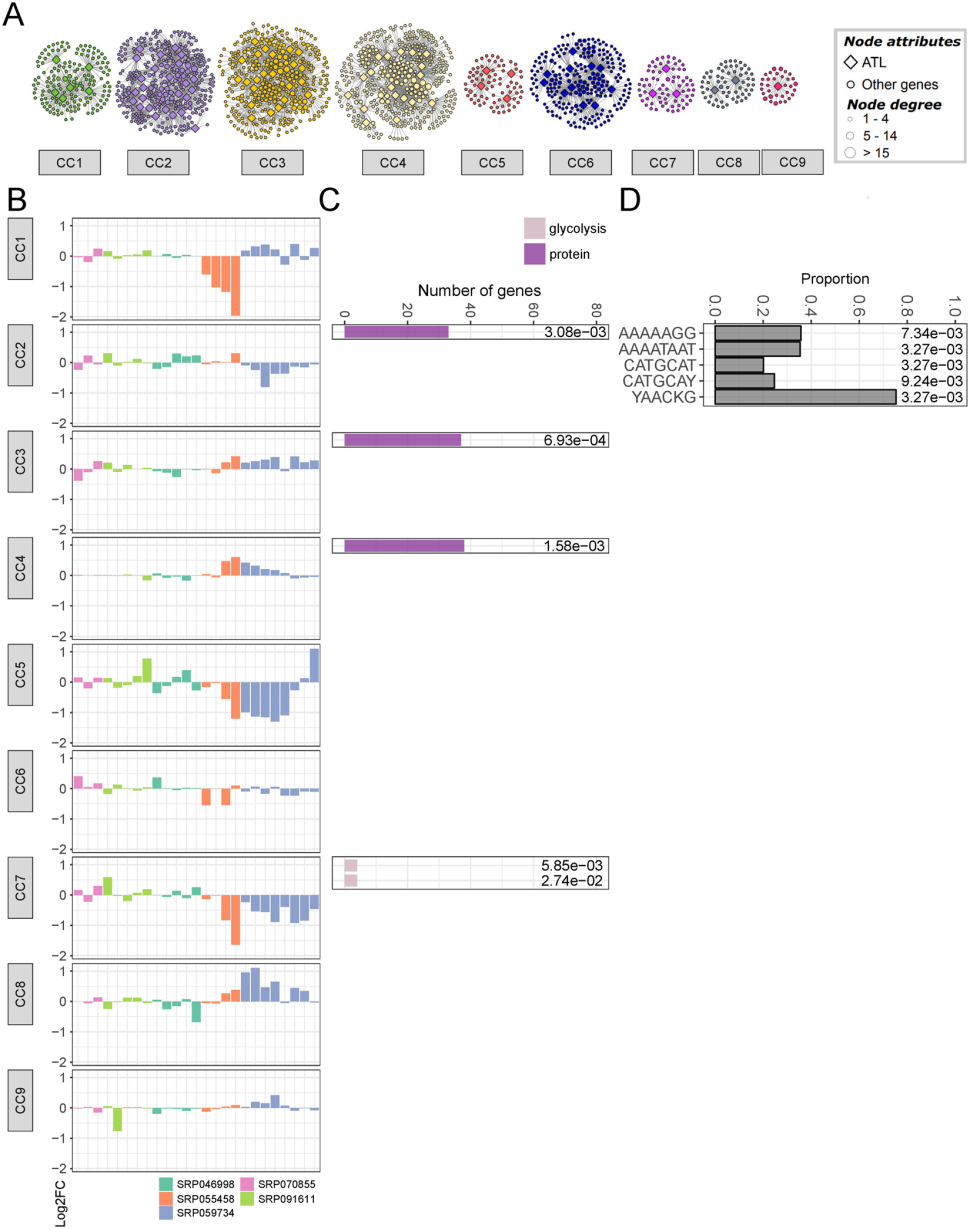
Combined grapevine ATL gene co-expression abiotic network. (**A**) The abiotic network was constructed using the average log2FC matrix comparing treatment vs control for the different abiotic stress-related conditions. Node color represents the assigned community cluster within the network. Diamonds designate grapevine ATL genes, whereas balls represent co-expressed genes. Ball size represents node degree, i.e. the number of connection for each node, as indicated. (**B**) Representation of gene expression in the 9 different abiotic community clusters according to the log2FC calculated for each condition. The different colors represent the different experimental datasets (see Supplementary Material for experiment details). (**C**) Distribution of enriched MapMan BIN categories in the 9 different abiotic community clusters. Only the first two levels of the MapMan BIN category hierarchy were shown. Bar color represents the different enriched MapMan BINs, while bar size represents the number of genes assigned to a given MapMan BIN. On the right of each chart is reported the FDR value calculated for each enrichment. (**D**) Enrichment of *cis*-acting regulatory elements (CAREs) in the 9 different abiotic community clusters. Bar size represents the proportion of co-expressed gene of a given cluster containing a given CARE, while on the right of each bar is reported the FDR value calculated for each CARE enrichment. FDR, false discovery rate.

**Figure 6:**
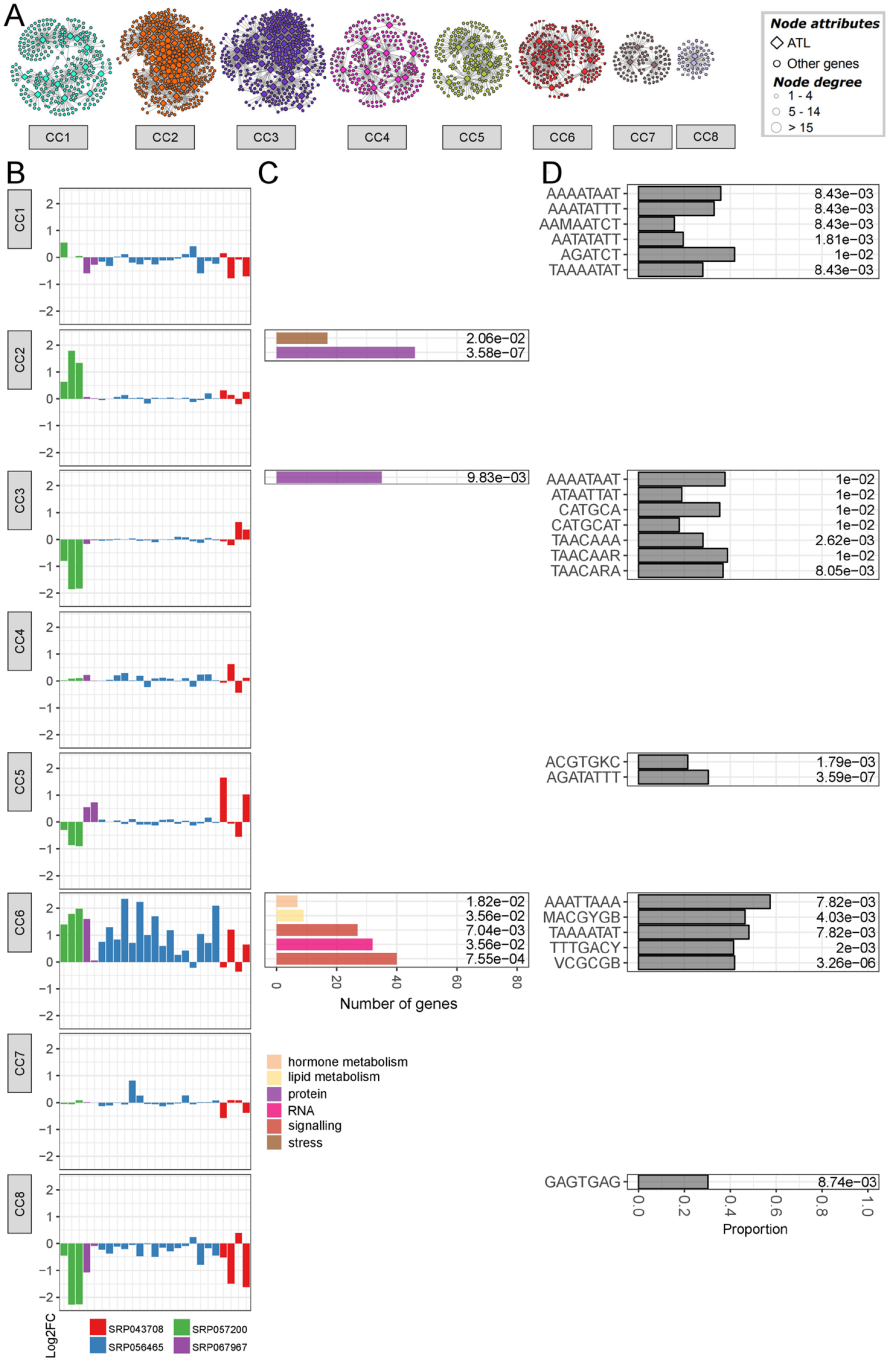
Combined grapevine ATL gene co-expression biotic network. (**A**) The biotic network was constructed using the average log2FC matrix comparing treatment vs control for the different biotic stress-related conditions. Node color represents the assigned community cluster within the network. Diamonds designate grapevine ATL genes, whereas balls represent co-expressed genes. Ball size represents node degree, i.e. the number of connection for each node, as indicated. (**B**) Representation of gene expression in the 8 different biotic community clusters according to the log2FC calculated for each condition. The different colors represent the different experimental datasets (see Supplementary Material for experiment details). (**C**) Distribution of enriched MapMan BIN categories in the 8 different biotic community clusters. Only the first two levels of the MapMan BIN category hierarchy were shown. Bar color represents the different enriched MapMan BINs, while bar size represents the number of genes of assigned to a given MapMan BIN. On the right of each chart is reported the FDR value calculated for each enrichment. (**D**) Enrichment of *cis*-acting regulatory elements (CAREs) in the 8 different biotic community clusters. Bar size represents the proportion of co-expressed gene of a given cluster containing a given CARE, while on the right of each bar is reported the FDR value calculated for each CARE enrichment. FDR, false discovery rate.

**Figure 7:**
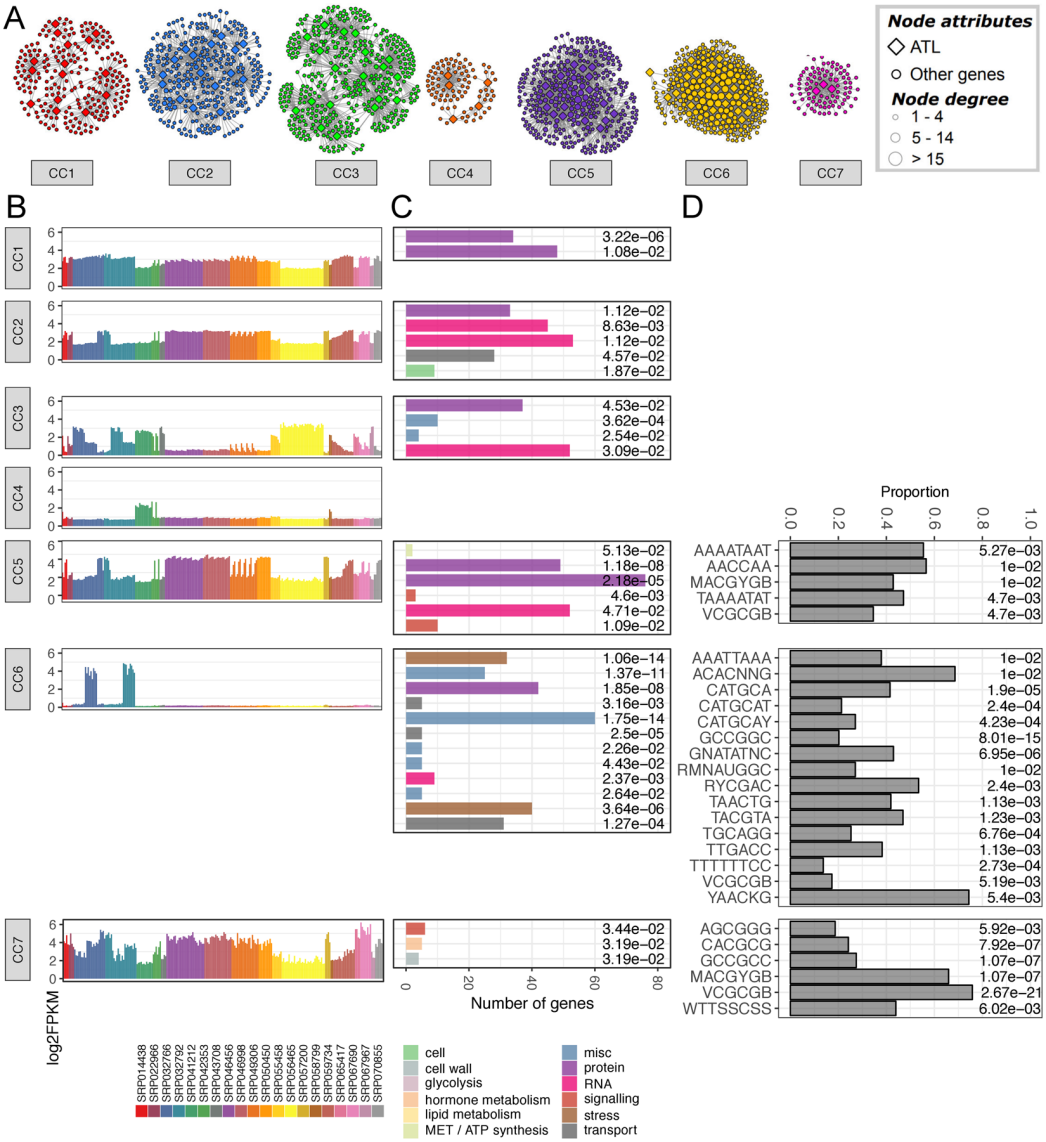
Combined grapevine ATL gene co-expression condition-independent network. (**A**) Condition-independent network was constructed using all RNA-Seq log2FPKM values (corresponding to transcript abundance) in the different samples. Node color represents the assigned community cluster within the network. Diamonds designate grapevine ATL genes, whereas balls represent co-expressed genes. Ball size represents node degree, i.e. the number of connection for each node, as indicated. (**B**) Representation of gene expression in the 7 different condition-independent community clusters according to the average log2FPKM calculated for each condition. The different colors represent the different experimental datasets (see Supplementary Material for experiment details). (**C**) Distribution of enriched MapMan BIN categories in the 7 different condition-independent community clusters. Only the first two levels of the MapMan BIN category hierarchy were shown. Bar color represents the different enriched MapMan BINs, while bar size represents the number of genes of assigned to a given MapMan BIN. On the right of each chart is reported the FDR value calculated for each enrichment. (**D**) Enrichment of *cis*-acting regulatory elements (CAREs) in the 7 different condition-independent community clusters. Bar size represents the proportion of co-expressed gene of a given cluster containing a given CARE, while on the right of each bar is reported the FDR value calculated for each CARE enrichment. FDR, false discovery rate.

To identify ATL-centric co-expression clusters that share multiple overlapping co-expressed genes, an additional step was undertaken to identify the community structure within the ATL condition-independent and condition-dependent GCNs (Figs 5–7A and Supplementary Table S7). Community clusters were built based on the profile of expression of ATL co-expressed genes in the different conditions considered in each dataset (abiotic, biotic, CI) (Figs 5–7B and Supplementary Figs S3–S4). Modules were mainly independent from one another in all three datasets, as shown by the low Pearson correlation coefficient (PCC) found between the community clusters (Supplementary Fig. S5). A strong correlation was found only for CC2 and CC5 in condition-independent dataset (PCC > 0.9), which were in turn correlated with CC7 (PCC > 0.7). This observation, especially for CC2 and CC5, indicate that these clusters may be part of a larger module. By contrast, in abiotic dataset, CC8 showed only a very slightly correlation with CC3 and CC4 (PCC > 0.5) and, in the same way, in biotic-related conditions, a slight correlation was observed between CC3 and CC8 (PCC > 0.7) and between CC4 and CC6 (PCC > 0.5).

The total number of nodes (genes) was 1,807 in seven condition-independent modules, 1,645 nodes in eight abiotic modules, and 1,836 nodes in nine biotic modules (Supplementary Table S8).

In the abiotic dataset, the mean number of genes per module was 182.8 (median = 157), compared to 229.5 (median = 247) in the condition-independent dataset and 258.1 (median = 259) in the biotic dataset. The modules in the condition-independent network featured a median of 16 ATL genes, compared to 10 in the biotic network and six in the abiotic network. All 96 grapevine ATLs^20^ were represented in the different condition-independent modules, whereas only 93 were represented in the abiotic and biotic modules. The three ATL genes missing from both stress-related networks included two whose absence was common to both datasets, namely *VviATL116* (VIT_09s0002g05140) and *VviATL158* (VIT_13s0019g01000), which were thus represented solely in the condition-independent GCN. These ATLs could therefore play a more substantial role in grapevine growth and development than stress responses. Interestingly, the condition-independent module CC7 and the biotic module CC6 solely contain genes representing the top18 defense-induced ATL proteins^20^. A comparison of these modules could therefore help to define the key features and functions of the grapevine ATLs that respond to biotic stress.

A possible sub- or neo-functionalization was previously proposed for ATL paralogs deriving from gene duplication (Ariani *et al*.^20^). Accordingly, the analysis of ATL paralog distribution within the different network modules showed that at least 70% of WGD-derived pairs and at least 65% of tandem duplication-derived pairs belong to separate community clusters, regardless of the dataset (Supplementary Table S9). Moreover, paralog pairs belonging to the same modules were not the same in all datasets, suggesting that new ATL functions may have arose through divergence in their expression pattern to respond to particular physiological conditions.

The clustering of co-expressed genes into modules can also reflect regulatory relationships found in a given physiological process. According to the principle of guilt by association (GBA), the identification of enriched functional categories within particular community clusters allows the putative functional annotation of genes with unknown functions thanks to their co-expression with well-characterized genes^41^. The analysis of BIN enrichment revealed that only BIN 29 (protein metabolism), and in particular BIN 29.5 (protein degradation), was commonly enriched in all three datasets (Figs 5–7C and Supplementary Fig. S6). Such enrichment validates the GBA principle and the robustness of the networks identified under different conditions because the modules were built using ATLs, whose principal molecular function is to facilitate proteolysis. Accordingly, the community clusters enriched for protein degradation corresponded to those containing the most ATL genes regardless of the conditions (Supplementary Table S8). Among all the community clusters enriched for BIN 29.5, more than 55% of the genes in this functional category were ATL genes. One exception was CC5 in the condition-independent dataset, where ATL genes represented only 45% of the genes related to protein metabolism (Supplementary Table S10). This cluster was also significantly enriched for the more general BIN 29 category, with more than 60 hits with an FDR < 1.0 (Fig. 7C and Supplementary Fig. S6). Indeed, in addition to the ATL family, CC5 included more than 20 E3 ubiquitin ligases belonging to other families (C3HC4-type zinc finger or Skp1–Cul1–F-box), as well as UBX domain-containing proteins, indicating this cluster was strongly associated with proteasome-mediated protein degradation.

Several BINs were specifically enriched in a single dataset. BIN 4 (glycolysis/cytosolic branch) was enriched only in abiotic CC7, which contained only 72 nodes in total, thus indicating significant glycolysis-related specialization, particularly for subnetworks *VviATL103* (VIT_19s0090g00400), *VviATL111* (VIT_02s0087g00420) and *VviATL138* (VIT_03s0038g03930) (Fig. 5C and Supplementary Fig. S6). These three ATL genes did not cluster in the condition-independent and biotic datasets, instead belonging to separate clusters. CC7 was characterized by the strong downregulation of genes under shaded conditions and during the night compared to daylight hours (Fig. 5B, Supplementary Fig. S3 and Supplementary Table S11). Similarly, biotic CC6, with 240 nodes including 10 ATL genes, was uniquely enriched for genes related to lipid degradation (BIN 11) (Fig. 6C and Supplementary Fig. S6). In the condition-independent GCN, the specifically enriched BINs were BIN 10 (cell wall modification), BIN 26 (miscellaneous enzyme families, particularly short chain dehydrogenase/reductases, peroxidases, *O*-methyltransferases and cytochrome P450), BIN 31 (vesicle transport) and BIN 34 (transport/nitrate/major intrinsic proteins) (Fig. 7C and Supplementary Fig. S6). These dataset-specific enrichments may reflect the specialized function of some ATL genes in particular processes, probably related to growth and development.

The co-enrichment of two or more BINs in a given cluster was also observed in several cases. BIN 17 (ethylene) and BIN 30 (signaling) were co-enriched in biotic CC6 and condition-independent CC7, although signaling-related genes in the biotic dataset were often associated with receptor kinases, whereas Ca^2+^ signaling was more relevant in the condition-independent dataset. Several Ca^2+^ signaling genes were also identified in biotic CC6 although the clustering was not statistically significant. Furthermore, these genes mainly encoded Ca^2+^-binding proteins, as observed in condition-independent CC7. Interestingly, both biotic CC6 and CI CC7 contained genes representing the top 18 pathogen-regulated ATL proteins^20^ that are strongly upregulated in response to biotic stress (Fig. 6B and Supplementary Fig. S4 and Supplementary Tables S12-S13). Moreover, both clusters included *VviATL156*, which is co-expressed with genes encoding Ca^2+^-binding proteins (BIN 30.3) and proteins involved in ethylene metabolism (BIN 17) in both the biotic and condition-independent datasets (Supplementary Table S4). VviATL156 could thus play an important role in signal transduction, in particular related to receptor kinases and Ca^2+^ sensing, leading to the regulation of ethylene-dependent responses.

### Enrichment of CAREs in the abiotic, biotic and condition-independent community clusters

Finally, CARE enrichment analysis was carried out as described above, this time focusing on the community clusters derived from the three different datasets (Figs 5–7D, Supplementary Fig. S7 and Supplementary Table S14). Overall, the biotic stress network featured the greatest number of modules enriched in CAREs, with five of the eight community clusters showing enrichment (CC1, CC3, CC5, CC6 and CC8) (Fig. 6D), whereas only three were enriched in the condition-independent dataset (CC5, CC6 and CC7) (Fig. 7D) and only one (CC2) in the abiotic dataset (Fig. 5D). Two CAREs were enriched in at least one community cluster in all datasets, i.e. RYREPEATGMGY2 (CATGCAT), which mediates ABA-induced transcription and repression by gibberellic acid, and the AT-hook DNA-binding motif of AHL20 (AAAATAAT), which is involved in the negative regulation of defense responses. The AHL20-binding motif was identified in the promoters of 123 genes in biotic CC1, including seven ATL genes, three of which were not modulated in response to biotic stress, whereas the other four were downregulated by pathogens^20^. The same motif was also found in 134 genes in biotic CC3, including 12 ATL genes, nine of which were modulated in response to biotic stress, mainly downregulated in response to necrotrophic pathogens^20^. Among the 109 genes containing the AHL20-binding motif in abiotic CC2, 14 ATL genes were identified, six of which are mainly downregulated under abiotic stress conditions^20^. Biotic CC1 and CC3 and abiotic CC3 were all enriched solely for BIN 29.5 (protein degradation), indicating that AHL-related CAREs may help to regulate the specificity of protein turnover. On the other hand, condition-independent CC5 was also enriched for the AHL20-binding motif, which was found in 194 genes, including 12 ATL genes. In the latter group, two genes were shared by biotic CC1 and CC3, three were shared with abiotic CC3, and seven were unique to condition-independent CC5 and therefore were unlikely to be involved in stress responses.

In addition to the common RYREPEATGMGY2 (CATGCAT) and AHL20 (AAAATAAT) motifs discussed above, most of the other CAREs were enriched specifically in a particular dataset. As well as the AHL20 motif, biotic CC1 and CC3 were also both enriched for AHL12-binding motifs (AAATATTT and ATAATTAT) and other AT-hook binding motifs involved in promoter repression, further suggesting that the downregulation of these two modules occurs in response to pathogens. In accordance with this hypothesis, three elements related to sugar-dependent repression were also identified in biotic CC3, namely AMYBOX 1 (TTAACARA), GAREAT (TAACAAR) and MYBGAHV (TAACAAA). On the other hand, biotic CC1 and CC5 were both enriched for CCA1 (CIRCADIAN CLOCK-ASSOCIATED 1) transcription factor binding sites (AAMAATCT and AGATATTT), which were recently shown to regulate the expression of some defense genes and to confer downy mildew resistance in *A*. *thaliana*^42^. Biotic CC1 was also enriched in the two-component response regulator ARR11-binding motif (AGATCT), which is involved in cytokinin-activated signaling pathways, and may account for the interactions between cytokinin-mediated responses and the circadian clock^43,44^. However, in biotic CC5, the CCA1 site was co-enriched with the ABA-responsive ACGTABREMOTIFA2OSEM element (ACGTGKC). Both motifs were present together in the promoters of 17 genes, reflecting the significant overlap among transcripts regulated by the circadian clock and ABA^45^, which might partly explain that many ABA-regulated responses to stress are influenced by circadian rhythms. Finally, biotic CC6 was enriched for the WBBOXPCWRKY1 motif (TTTGACY), which, contrary to the CAREs found in CC3, is thought to be required for sugar-dependent induction^46^.

## Discussion

The ubiquitinylation system is an important regulatory mechanism for a broad range of developmental processes and abiotic and biotic stress responses in plants^47^. E3 ubiquitin ligases, which are the most numerous and diverse components of the ubiquitination pathway^48^, play a central role in the determination of substrate specificity. A recent survey in grapevine identified 96 members of the ATL family, a class of E3 ubiquitin ligases containing a RING-H2 domain^20^. Phylogenetic analysis revealed that new ATL genes have arisen and have been retained mainly following whole-genome duplication (WGD), suggesting sub-functionalization has driven their specialization. This hypothesis was first supported by the diverse expression profiles of ATL genes in different physiological contexts, from growth and development to abiotic and biotic stress responses (Ariani *et al*.^20^). Here, we found that the different paralog ATL pairs, derived from whole-genome or tandem duplication, mainly belong to separate community clusters in the different networks (or even only one paralog is found in the network). Moreover, the few pairs belonging to the same modules, likely reflecting a similar function in a given condition, are different depending on the dataset. For instance, *VviATL156* (VIT_05s0077g01970) belongs to the same module as its paralog *VviATL155* (VIT_07s0005g00710) in abiotic- and CI-related datasets, while it belongs to a different module in the biotic dataset, where it seems to play a major and likely unique role (see below). This strongly sustains the importance of ATL sub-functionalization and/or neo-functionalization deriving from gene duplication, which might confer in grapevine specific adaptation to particular conditions.

To investigate the potential functions of grapevine ATL genes, correlation analysis was carried out to identify co-regulated gene pairs involved in different biological processes. Further information was gained by looking for functional category enrichment among the co-expressed ATL genes, and establishing cluster communities in different datasets based on the GBA principle, which postulates that two biological entities with similar quantitative behaviors may have similar functions^41^. Finally, promoter analysis was carried out on the entire grapevine ATL family and on specific community clusters retrieved from all datasets. Genes with similar expression profiles and biological functions are likely to be regulated by the same transcription factors, so the presence of similar CAREs for particular transcription factors within subnetworks or community clusters is a reliable strategy to define putative network regulators that are active under specific conditions^49–51^. Ubiquitinylation regulates a wide range of physiological processes, which must be fine-tuned on a spatial and temporal scale^1,3^. Information about the expression and coordinated action of E3 ligases may therefore help to identify upstream signaling components or downstream substrates of ubiquitinylation, allowing their further experimental testing to confirm their role in plant stress responses.

### Co-expression networks can identify the potential roles of grapevine ATL genes

The application of network-based approaches including GCNs in several grapevine studies has led to a better understanding of coordinately regulated genes involved in many aspects of grapevine development and metabolism^26^. Similar to previous grapevine GCN studies^23,24,52–54^, we began our analysis by first establishing a condition-independent GCN using publicly available RNA-Seq data to provide a global overview of gene-to-gene relationships under various experimental conditions (Supplementary Material). In this study, we chose RNA-Seq data rather than the more widely available Nimblegen microarray datasets, which have often been used for GCN analysis, to ensure that our downstream GCN analysis was more reliable, in particular due to the potential for cross-hybridization among microarray probe sets^55^. Furthermore, microarrays have now been largely supplanted by RNA-Seq as the preferred basis for transcriptome analysis. We used GCC values to quantify ATL gene co-expression relationships because recent surveys show that this method overcomes the shortcomings of popular co-expression metrics, such as Pearson’s and Spearman’s correlation coefficients, when focusing on regulatory genes using RNA-Seq data^56^.

Condition-independent datasets can potentially mask key co-expression relationships that are evident only under specific conditions^26,57^, so we included two condition-dependent datasets representing abiotic and biotic stress conditions. This enabled us to fully explore potentially interesting regulatory relationships that are enhanced by specific forms of stress. This choice was supported by our earlier expression meta-analysis, showing that many grapevine ATL genes are differentially expressed in response to stress^20^.

### Functional category and CARE enrichment across datasets suggest a close relationship between ATL proteins, transcription factors and hormones

The analysis of GCNs and CAREs suggested the preferential involvement of some ATL proteins in the regulation of protein metabolism, transcription and signaling (particularly receptor kinases and Ca^2+^ signaling) in both the condition-independent and stress-related datasets. For example, several GCN modules, regardless of the experimental conditions, were enriched for BIN 29.5 (protein degradation). It would be tempting to assume that this reflected the presence of numerous ATL genes in the GCNs that were built using the top 100 co-expressed genes. However, at least half of the genes annotated in this functional category encoded non-ATL E3 ubiquitin ligases or other proteins, including subtilases, ubiquitin-related proteins, proteasome subunits, E2 ubiquitin-conjugating enzymes, cysteine/aspartate/serine proteases, metalloproteases and AAA-type ATPases. These clusters confirmed the role of some ATL proteins in regulatory processes requiring protein degradation, but processes highlighted by the clusters that were not enriched for protein degradation functions indicate that the ATL proteins in these clusters have more diverse roles, e.g. biotic CC6 and condition-independent CC7 were both enriched for BIN 30 (signaling), suggesting that ATL genes in these clusters regulate cellular functions other than protein degradation.

The involvement of ATL proteins in diverse processes is documented by the functional annotation of genes in their co-expression networks, most of which were classified as transcription factors or signaling components. The identification of many genes encoding transcription factors in the ATL co-expression networks suggests a strong correlation between ATL genes and the main transcription factor families (Supplementary Fig. S8). More specifically, eight ATL subnetworks were specifically enriched for WRKY transcription factors and the corresponding ATL proteins tended to be involved in pathogen defense. The UPS modulates the abundance of transcription factors to effect changes in gene expression required to respond to stressful environmental conditions. For example, the dehydration-response element binding protein (DREB) 2 A, which regulates the expression of many genes induced by drought and salt stress^58,59^, is in turn regulated by the RING E3 ligases DRIP1 and DRIP2^60,61^. Thus, the transcription factors in the grapevine ATL subnetworks may be the targets of the corresponding ATL proteins. The analysis of CAREs in the promoters of ATL genes and the corresponding GCN support the view that the transcription of a given ATL gene and its co-regulated genes is often regulated by common transcription factors.

The close relationship between ABA and ubiquitinylation is well documented, and ubiquitinylation plays an important role in ABA signaling, from the ubiquitinylation of ABA receptors to the regulation of signaling molecules^62^. The phenotype of the *A*. *thaliana* ATL43 deletion mutant suggests that ATL family E3 ligases may play a role in the ABA response^6^. The analysis of co-expressed genes and CAREs shared among grapevine ATL promoters strongly supports this hypothesis. Moreover, responses to drought stress and pathogens partially overlap, with ABA as a positive stimulator^63^. Accordingly, whatever the conditions and dataset considered, many grapevine ATL genes showed a close relationship with ABA responses, in terms of co-expressed gene frequency, ABA-related functional enrichment in ATL subnetworks and ABA-responsive CAREs. Nevertheless, none of the community clusters were enriched for an ABA-related functional category, although several clusters in the condition-independent and stress-dependent datasets were enriched for ABA-responsive CAREs. Such elements were also found in the ATL gene promoters, suggesting that ATL genes and co-expressed non-ATL genes may be commonly regulated at the transcriptional level once the ABA signal is transmitted to the nucleus. Together these results indicate that ATL-mediated ubiquitinylation may play a role in transducing the ABA signal rather than regulating ABA metabolism or perception. In this context, some ATL proteins may play a positive role in ABA signaling, e.g. AtATL43^6^, whereas others may be negatively regulated by ABA, e.g. AtATL78, the expression of which is repressed by ABA/drought but induced by cold stress^17^. Such differential regulation may ensure a specific cellular response.

In line with the recent demonstration that *A*. *thaliana* ATL15 is regulated by sugar^7^, it is notable that in several of our community clusters (e.g. biotic CC3 and condition-independent CC6), the ABA-related CAREs were co-enriched along with CAREs related to sugar-mediated repression (TACGTA, TAACARA, TAACAAR, TAACAAA). ABA may play an important regulatory role in the sugar supply and/or osmoregulation under both biotic and abiotic stress conditions^63^. Moreover, many genes are co-regulated by sugar (glucose) and ABA, including key regulators of ABA signaling and a diverse set of genes involved in signal transduction, transcription, stress responses, and metabolism^64^, the same categories we observed in ATL subnetworks across all three datasets. Furthermore, several genes involved in ethylene-mediated gene expression are also co-regulated by ABA and glucose, identifying regulatory points for three-way interactions among these regulators of growth and stress responses^65,66^.

Another well-known relationship between hormones and proteasome-mediated degradation is the regulation of gibberellin-dependent responses by DELLA protein degradation^67^. DELLA proteins belong to one of the eight subfamilies of GRAS transcription factors. *A*. *thaliana* has five DELLA proteins, namely GAI, RGA, RGA-LIKE1 (RGL1), RGL2 and RGL3, which function as repressors of gibberellin-responsive plant growth. Following the perception of GA_3_, the E3 ubiquitin ligase SKP1-Cullin-F-box (SCFSLY1/GID1) complex polyubiquitinylates DELLA proteins and triggers their degradation by the 26 *S* proteasome^68^. Surprisingly, none of the GRAS transcription factors co-expressed with grapevine ATL genes belong to the DELLA subfamily, suggesting that other GRAS subfamilies may be regulated by proteasome-mediated degradation in grapevine. Accordingly, among the 12 GRAS transcription factors enriched in the different ATL subnetworks from the condition-independent dataset, four deserve particular attention because they belong to the newly identified subfamilies GRAS8 and GRASV, both specific to grapevine and putatively involved in ripening and the response to *B*. *cinerea*^37^. The co-expression of GRAS8 and GRASV with grapevine ATL genes suggests a novel pathway for GA_3_ signal transmission, in which ATL E3 ligases are regulated by GA_3_ in accordance with the presence of GA_3_-response elements in ATL promoters, and the ATL proteins would in turn regulate the GRAS transcription factors. Given that *VviATL93* is co-expressed with both GRASV1c and GRASV2a, and was previously reported to be predominantly upregulated in juvenile samples including the tendril and inflorescence^20^, this ATL deserves particular attention because by regulating grapevine specific GRAS transcription factors it could facilitate the specific GA_3_ responses observed during grapevine development. The transmission of the GA_3_ signal in the floral genetic pathway promotes flowering in *A*. *thaliana* but inhibits floral meristem development in grapevine, thus leading to the formation of a tendril as a new organ^69^.

### Grapevine ATL proteins during abiotic stress

Several abiotic community clusters (CC1, CC5, CC6, CC8 and CC9) showed no enrichment for particular BIN categories or CAREs, but this does not necessarily mean the corresponding ATL proteins have no specific roles under abiotic stress conditions. Moreover, CC3 and CC4 were not enriched for CAREs but both were enriched for BIN 29.5 (protein degradation). Overall, there was not much clustering of ATL genes and corresponding co-expressed genes in the abiotic dataset, resulting in low connectivity and low biological relevance. This may reflect the lower number of conditions included in the abiotic dataset compared to the biotic dataset. In future, more RNA-Seq datasets representing diverse forms of stress (e.g. UV or cold stress) may be required to yield more comprehensive and informative GCNs under abiotic stress conditions. Alternatively, ATL proteins may generally be less involved in abiotic stress responses than biotic stress responses, such that broadening the range of conditions would not increase the number of differentially expressed genes and corresponding networks.

Despite the low number of enrichments, abiotic CC7 was characterized by the significant downregulation of ATL genes and co-expressed genes related to day/night transitions and shading, and this cluster was also uniquely enriched for BIN 4 (cytosolic branch of glycolysis). Genes involved in respiratory metabolism are often influenced by shading. In particular, the expression of enzymes associated with the cytosolic branch of glycolysis is downregulated in the shade, including enolase, glyceraldehyde 3-phosphate dehydrogenase (GAPDH), phosphoenolpyruvate carboxylase (PEPC), pyruvate kinase and UDP-glucose pyrophosphorylase^70^. The key step in glycolysis is catalyzed by pyruvate kinase, which converts phosphoenolpyruvate to pyruvate. In human cells, pyruvate kinase M2 (PKM2) is the unique substrate of the ubiquitin E3 ligase Parkin. The latter regulates glycolysis (and thus cell metabolism in general) via the ubiquitinylation of PKM2 and its subsequent degradation^71^. A similar process occurs in plants, where ubiquitinylation following the phosphorylation of specific residues is essential for the degradation of cytosolic pyruvate kinase via the proteasome pathway^72^. However, the enzymes involved in the ubiquitinylation of cytosolic pyruvate kinase in plants are still unknown. Our data suggest that ATL E3 ligases may fulfil this role, in particular VviATL138 (VIT_03s0038g03930), VviATL23a (VIT_18s0001g01060), VviATL148 (VIT_14s0128g00120) or VviATL112 (VIT_19s0015g01000), because their subnetworks are enriched for glycolysis-related genes. VviATL138 (VIT_03s0038g03930) is an interesting candidate for further investigation because it belongs to abiotic CC7, together with three pyruvate kinase genes, which thus represent putative VviATL138 substrates.

VviATL23a (VIT_18s0001g01060) is also particularly interesting because it is predicted to be localized in the mitochondria^20^, and cytosolic glycolytic enzymes (including pyruvate kinase) are physically associated with the outer mitochondrial membrane^73^. In addition to glycolysis (BIN 4), the VviATL23a abiotic stress-related subnetwork is also enriched for BIN 5 (fermentation). Fermentation pathways in yeast include enzymes with the highest proportion of lysine residues modified by ubiquitinylation, particularly pyruvate decarboxylases and alcohol dehydrogenases^74^, and these genes are also present in the VviATL23a subnetwork. VviATL23a may therefore fine-tune the irreversible reactions catalyzed by these enzymes under different metabolic and environmental conditions.

### Biotic stress networks highlight an important role for grapevine ATL proteins in responses to pathogens

A previous transcriptomic survey revealed that 64 grapevine ATL genes are differentially expressed in response to biotic stress, including 12 that are strongly induced by biotrophic pathogens such as powdery and downy mildew, and also by necrotrophic pathogens^20^. Detailed analysis of the co-expression subnetworks of these TOP 12 “pathogenesis-related” ATLs^20^ revealed enrichments in functional categories such as the regulation of transcription (BIN 27.3, particularly WRKY transcription factors), signaling (BIN 30, particularly Ca^2+^ signaling (BIN 30.3) and receptor kinase signaling (BIN 30.2)), as well as lipid degradation (BIN 11.9.3) and ethylene metabolism (BIN 17.5). All these functional categories strongly support a role for at least some ATL proteins in the regulation of defense-related signal transduction, via the activation of transcription factors, receptor kinase cascades and calcium-dependent signaling.

Interestingly, BIN 29 (protein metabolism) was enriched only in one pathogenesis-related ATL subnetwork (*VviATL91*, VIT_13s0019g01980), suggesting that ATLs involved in plant immunity mainly regulate defense responses in pathways requiring protein degradation for the fine-tuned control of signal transduction rather than by targeting proteins for proteasome-mediated degradation. This could be achieved by target protein mono-ubiquitinylation, which can function as a molecular switch in certain regulatory processes, including signal transduction, transcriptional regulation, chromatin remodeling and DNA repair^75–77^. For example, *A*. *thaliana* histone H2B is mono-ubiquitinylated by two RING E3 ligases (*HISTONE MONOUBIQUITINATION1* [*HUB1*] and *HUB2*) and a recent study revealed that *A*. *thaliana* HUB1 regulates defense against necrotrophic fungal pathogens^78^, thus linking key target protein mono-ubiquitinylation and plant defense responses.

In an alternative hypothesis, target protein function could also be altered by lysine-63 linked polyubiquitinylation, which has non-proteolytic functions such as protein activation and endocytosis^79^. One of the best model plasma membrane cargoes regulated by endocytosis in plants is the receptor-like kinase (RLK) family, in particular leucine-rich repeat (LRR) RLKs, as observed for BRASSINOSTEROID INSENSITIVE 1 (BRI1)^80,81^ or the well-known receptor for bacterial flagellin, FLAGELLIN SENSING 2 (FLS2)^82^, which was recently shown to be internalized via clathrin-dependent endocytosis^83^. Intriguingly, biotic CC6, which includes nine of the TOP12 pathogen-related ATL genes induced in response to pathogen infection^20^, also contains 27 annotated RLKs representing more than 10% of the genes in the module, thus highlighting a strong relationship between ATLs and RLKs in response to pathogens. Moreover, the translation inhibitor cycloheximide probably blocks the *de novo* synthesis of proteins that contribute to the induction of FLS2 degradation^84^. The ATLs in biotic CC6 are strongly upregulated, suggesting that ATLs in this module may be required for the ubiquitinylation of RLKs, leading to their internalization.

In contrast, some ATL proteins may play a negative role in grapevine defense responses and would thus be downregulated by biotic stress. Accordingly, the co-expressed genes in biotic CC1 and CC3 are enriched in motifs related to the negative regulation of defense responses, namely the AHL12 and AHL20 binding motifs^39^. The potential relevance of AHL-related CAREs and transcription factors in the negative regulation of gene expression is supported by recent studies demonstrating the role of AtAHL12 as a transcriptional repressor in tobacco, and the consistent enrichment for AHL CAREs in the genes negatively co-regulated with AHL proteins^39^. In the context of biotic stress, *A*. *thaliana* AHL proteins such as AtAHL20 are involved in the negative regulation of plant immunity, whereas others (e.g. AtAHL15, AtAHL19 and AtAHL27) block gene expression induced by pathogen-associated molecular patterns in transient protoplast assays^85^. In line with the possible role of these regulatory elements, both CC1 and CC3 contain ATL genes that are also coordinately downregulated in response to pathogens, suggesting a pathogen-responsive regulatory network in grapevine targeted by AHL proteins. Moreover, biotic CC3 is also enriched in CAREs responsible for promoter repression by sugars (TAACARA, TAACAAR and TAACAAA), indicating that the expression of the genes in this module is downregulated by several pathways. Interestingly, sugars could also play the opposite role, given that biotic CC6 (which mainly contains genes that are induced by pathogens) is enriched for the WBBOXPCWRKY1 motif (TTTGACY), which may be required for sugar-dependent induction^46^. It would be interesting to investigate whether sugars differentially regulate diverse ATL genes and related genes to ensure the induction of defense responses to necrotrophic pathogens. Moreover, because glucose and fructose may have distinct roles in defense against *B*. *cinerea*^86^, it would be interesting to determine whether different sugars are responsible for the differential regulation of ATL GCNs.

### VviATL148 and VviATL156: putative key regulators of biotic stress responses in grapevine

**Figure 8:**
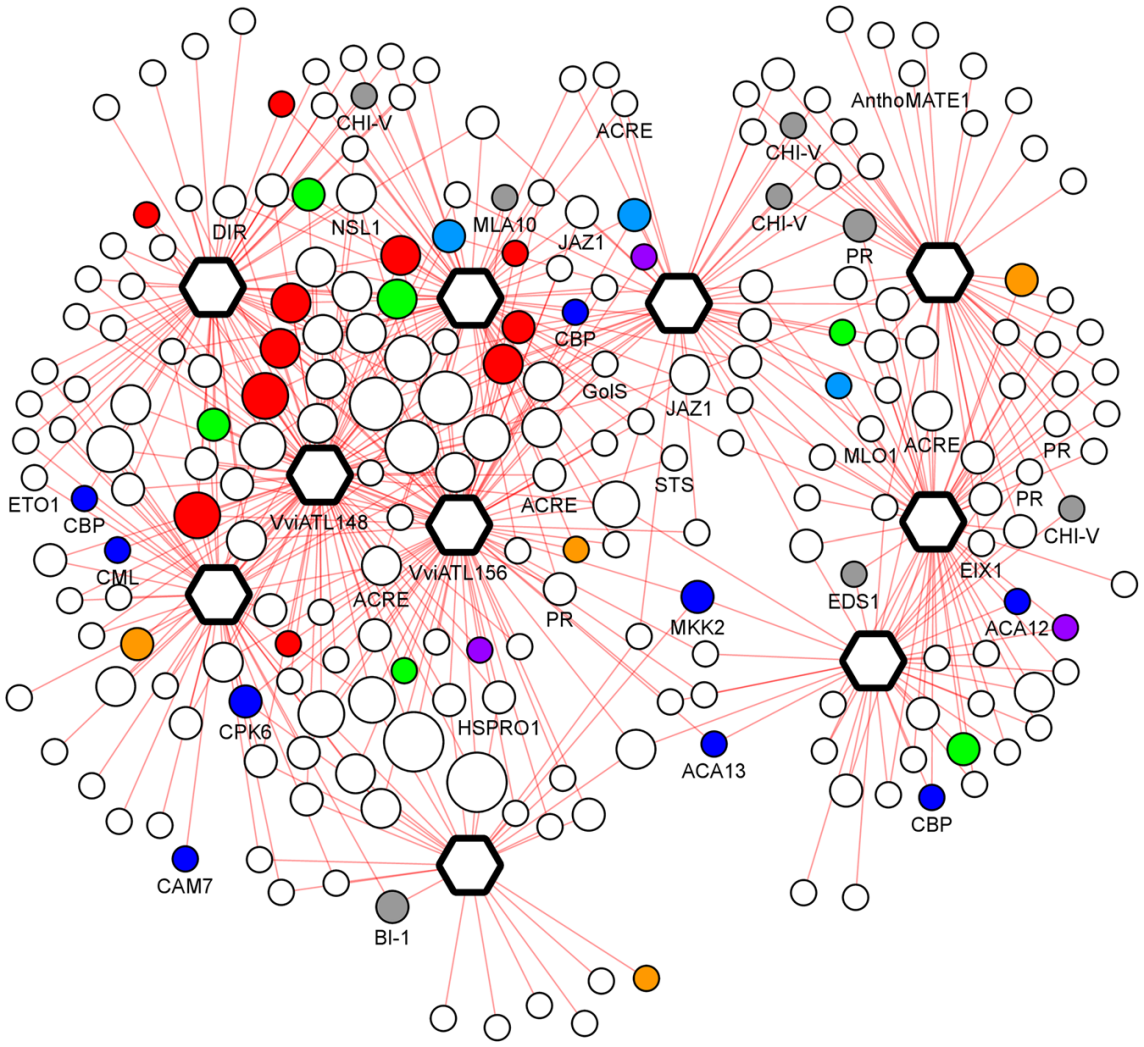
Biotic-specific CC6 module. Hexagons represent ATLs and circles represent ATL co-expressed genes (nodes). Node color depicts assigned functions of co-expressed genes involved in plant-pathogen interaction (grey) or calcium signaling (blue), or indicates the main TF families (red, WRKY; blue, MYB; green, AP2/ERF; purple, C2H2; yellow, bZIP; orange bHLH). Node size represents the number of TFs connected with a given ATL.

Because our final goal was to identify candidates for future functional analysis and potential biotechnological applications, we focused on specific features emerging from the co-expression networks of a few selected ATL proteins in biotic CC6, which may be useful for the development of improved pathogen resistance (Fig. 8). For example, the GCC-box motif described above was enriched in the defense-related *VviATL148* (VIT_14s0128g00120) subnetwork. Remarkably, as many as 51 of the 100 co-regulated genes in the biotic dataset (only 68 with an assigned ontology) represent the signal transduction, transcription factor or protein turnover categories. Recurrent annotations in the biotic subnetwork include Ca^2+^-regulated proteins, receptors kinases, WRKY transcription factors and ethylene-responsive transcription factors. VviATL148 is the grapevine ATL with the greatest similarity to *A*. *thaliana* ATL31 and its closest homolog ATL6, which play key roles in the carbon/nitrogen balance^87,88^ as well as defense against pathogens. Transgenic plants overexpressing ATL31 or ATL6 displayed increased resistance to virulent *P*. *syringae* pv. *tomato* (Pst) DC3000, whereas the *atl31atl6* double knockout mutant was more susceptible to this pathogen than wild-type plants^19^. There is a close transcriptional correlation between ATL31 and the transcription factors WRKY53 and WRKY33, which are involved in defense responses^19,89^, and finally both WRKY33 and WRKY53 have been shown to regulate *ATL31* gene expression during defense responses^90^. Several grapevine genes that are highly similar to *A*. *thaliana* WRKY33 and WRKY53 are also co-regulated with *VvATL148* in the biotic dataset (GCC > 0.8) suggesting the signaling cascades are conserved. The promoters of genes co-expressed with *VviATL148* are enriched for motifs regulated by hormones (ABA, JA and ethylene), Ca^2+^, and WRKY transcription factors. For example, the CACGTG and ABRERATCAL motifs, found in defense-related and ABA-regulated genes, were identified in 36 and 53 ATL promoters, respectively. The CGCGBOXAT and WBBOXPCWRKY1 elements were likewise present in 45 and 46 promoters, respectively (FDR < 0.17E-02 and 0.02E-02, respectively).

One of the strongest correlations in the biotic *VviATL148* subnetwork was with *VviATL156* (VIT_05s0077g01970), which is strongly induced in resistant *V*. *riparia* following infection with *P*. *viticola*, and also in grapevine cultivars infected with *E*. *necator*. Moreover, it is differentially modulated in late defense responses against the pathogens *Eutypa lata* and *Neofusicoccum parvum*. Homology searches revealed that VviATL156 is most closely related to *A*. *thaliana* ATL2 and the poplar ATL protein RHE1, both of which are involved in responses to elicitors and pathogens^12,91^. These observations indicate that VviATL156 regulates protein targets involved in defense by ubiquitinylation. As shown for VviATL148, the *VviATL156* gene is highly co-regulated (GCC ≥ 0.8) with the genes encoding three ethylene-responsive AP2/ERF transcription factors, one MYB protein, one bHLH protein and nine WRKY proteins, seven calcium signaling components, and seven receptor kinases. Accordingly, the biotic *VviATL156* subnetwork was significantly enriched for BIN 27.3 (regulation of transcription) and BIN 30.3 (calcium signaling). The same BINs were also enriched in the condition-independent dataset, thus confirming the robustness of the association between VviATL156, signaling and transcription regardless of the conditions. VviATL156 showed high connectivity in both biotic CC6 and condition-independent CC7, which share a common enrichment for BIN 17.5 (ethylene) and BIN 30 (signaling), thus reinforcing its role in the global regulation of these functional categories. Although it is unclear whether VviATL156 acts upstream or downstream of Ca^2+^, CARE analysis revealed that the *VviATL156* promoter and the promoters of co-expressed genes were significantly enriched for the CGCGBOXAT and ABRERATCAL motifs that are regulated by Ca^2+^ and calmodulin^92^. This suggests that genes in the *VviATL156* subnetwork are commonly regulated by Ca^2+^ and calmodulin, and indeed two calmodulin-like genes are present in the subnetwork. On the other hand, the *VviATL156* promoter contains 27 putative WRKY sites which are potential targets for the eight WRKY transcription factors shared by the biotic VvATL156 subnetwork and the biotic CC6 module (WRKY6, WRKY11, WRKY15, WRKY18, WRKY33, WRKY40, WRKY53 and WRKY72). WRKY11, WRKY18, WRKY40 and WRKY53 are involved in basal plant defense responses, either positively or negatively^93–95^. It is thus tempting to assume that VvATL156 may play a general role in grapevine defense responses following infection with diverse microbes rather than regulating a specific grapevine–pathogen interaction. Accordingly, the expression of *VviATL156* is strongly induced in grapevine plants infected with *B*. *cinerea* but also *E*. *necator* and *P*. *viticola*, regardless of the resistance or susceptibility of the genotype^22,32,33,96^. Nevertheless, following infection with *P*. *viticola*, *VviATL156* is upregulated more rapidly and more strongly in resistant *V*. *riparia* than susceptible *Vitis vinifera*^22^, suggesting an important role for ATL proteins in the establishment of resistance. The targets of VviATL156 may include WRKY11 because this transcription factor is targeted by the E3 ligase EIRP1 in *V*. *pseudoreticulata*, promoting its degradation. WRKY11 negatively regulates basal defense mechanisms^93^, suggesting that EIRP1 promotes resistance by targeting this transcriptional repressor of defense responses. This hypothesis is further supported by the predicted localization of VviATL156 in the nucleus^20^.

## Methods

### Meta-analysis of grapevine RNA-Seq transcriptome datasets

The latest publicly available RNA-Seq datasets deposited in the NCBI Sequence Read Archive (http://www.ncbi.nlm.nih.gov/sra) were downloaded and processed according to previously established pipelines^24,25^ with modified read mapping and summarization steps using newer state-of-the-art tools. Briefly, filtered single-end or paired-end reads from Trimmomatic v0.36^97^ were aligned against the 12x grapevine reference genome^98^ using HISAT2 v2.0.5^99^ with default settings, and count summarized using featureCounts^100^ with the grapevine 12 × v1 (http://genomes.cribi.unipd.it/) reference annotation. Gene expression, expressed as reads/fragments per kilobase of transcript per million mapped reads (RPKM/FPKM, referred to as FPKM herein), were calculated with DESeq2^101^. A total of 654 conditions containing non-averaged biological was subsequently averaged and log2 transformed resulting in a final gene expression matrix comprising 236 conditions and 29,970 genes (DatasetCI). Contrast matrices in the form of log2 fold-change differences between two conditions (e.g. treatment vs control) were also constructed for abiotic (DatasetA) and biotic (DatasetB) stress datasets, both containing 25 contrasts each, using DESeq2 with previously established pipelines^102^.

### Gene co-expression network analysis

Three gene co-expression networks were constructed using the GCC as the co-expression similarity index in the rsgcc package^56^. One condition-independent network (DatasetCI) and two condition-dependent/specific networks (using DatasetA and DatasetB) were queried separately for the top 100 co-expressed gene partners (ranked by descending GCC values) with each of the 96 ATLs^20^. The statistical significance of the GCC values was estimated by determining the 90^th^, 95^th^ and 99^th^ percentiles of a background GCC distribution of all gene pairs (1,000*999*0.5) from a single random sampling of 1,000 genes^103^. This procedure was carried out 100 times and the average GCC within each percentile category represented robust estimates of the false positive rate at 10%, 5% and 1%, respectively. In the corresponding condition-independent, abiotic and biotic networks, individual ATL-centered co-expression clusters were also merged, and nodes with < 2 connecting edges were discarded, before partitioning into communities of densely connected nodes using GLay^104^, prior to visualization in Cytoscape v3.0^105^.

### Functional enrichment and CARE analysis

Enrichment p-values, based on a FDR-adjusted hypergeometric distribution of MapMan BIN categories was determined using 29,424 genes with annotated BIN categories as previously described^25,54^. Similarly, enrichment p-values (FDR) for selected CAREs of 6–8-mer were determined using 29,839 candidate promoter sequences as the background. MapMan BIN categories and CAREs were considered significantly enriched within the corresponding ATL-centered co-expression clusters and community clusters with FDR thresholds of <0.05 and <0.01, respectively. Additional CARE analysis to determine the positional bias score (Z-score) based on combined occurrence frequencies and position information^38^, were used to rank biologically relevant CAREs within the ATL gene promoters.

## Electronic supplementary material

Supplementary figures

Dataset 1

Dataset 2
