# Supplementary material for: Co-expression network analysis and cis-regulatory element enrichment determine putative functions and regulatory mechanisms of grapevine ATL E3 ubiquitin ligases
Source: Sci Rep. 2018 Feb 16;8:3151. doi: 10.1038/s41598-018-21377-y (PMC5816651; doi:10.1038/s41598-018-21377-y)
Supplement: Supplementary file 1 — Supplementary figures [file 41598_2018_21377_MOESM1_ESM.doc]

# Co-expression network analysis and cis-regulatory element enrichment determine putative functions and regulatory mechanisms of grapevine ATL E3 ubiquitin ligases

**Darren C.J. Wong^1,†^, Pietro Ariani^2^, Simone Castellarin^1^, Annalisa Polverari^2,*^, Elodie Vandelle^2,*^**

^1^ Wine Research Centre, University of British Columbia, 2205 East Mall, Vancouver, BC V6T 1Z4, Canada

^2^ Dipartimento di Biotecnologie, Università degli Studi di Verona, Verona, 37134, Italy

**^†^** present address: Ecology and Evolution, Research School of Biology, The Australian National University, Acton ACT 2601, Australia

^*^corresponding authors: Annalisa Polverari (annalisa.polverari@univr.it); Elodie Vandelle (elodiegenevieve.vandelle@univr.it)


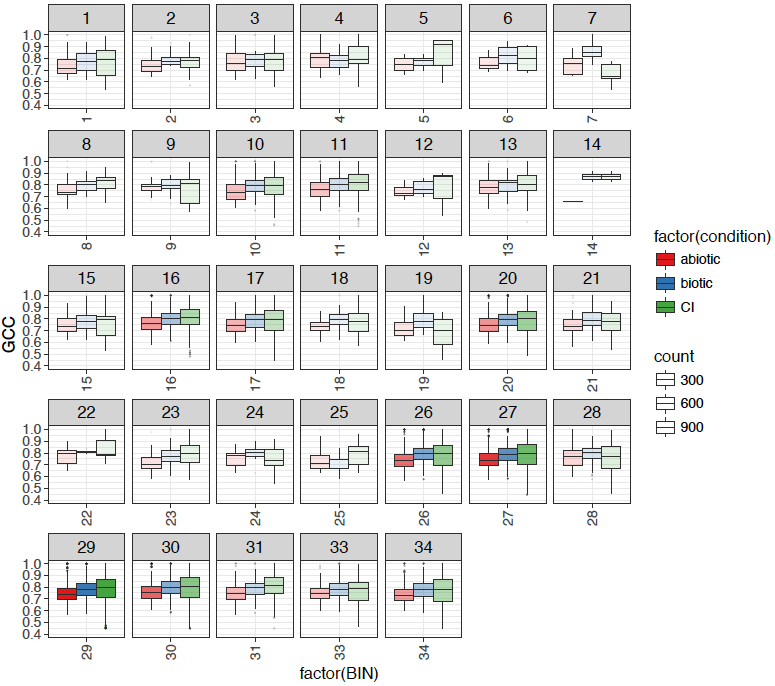


**Supplemental Figure S1. Distribution of GCC correlation values in the top 100 genes co-expressed with grapevine ATLs in three different datasets.** The number indicated on the top of each box indicates the BIN according to MapMan BIN annotation. The color indicates the dataset used for co-expression analysis (red, abiotic; blue, biotic; green, condition-independent), while the opacity represents the number of genes belonging to each MapMan BIN, with darker shading indicating a higher number of genes compared to other BINs

**Supplemental Figure S2. Distribution and frequency of MapMan BINs in the top 100 genes co-expressed with grapevine ATLs in three different datasets.** The number indicated on the top of each box indicates the BIN according to MapMan BIN annotation. The color indicates the dataset used for co-expression analysis (red, abiotic; blue, biotic; green, condition-independent).

**Supplemental Figure S3. Representation of gene expression in grapevine ATL community clusters in abiotic stress conditions.** Violin plots summarize gene expression in the 9 different networks with the black point designating the mean of log2FC calculated for each condition indicated on the X-axis (factor). The different colors represent the different community clusters (CCs), from CC1 to CC9.

**Supplemental Figure S4. Representation of gene expression in grapevine ATL community clusters in biotic stress conditions.** Violin plots summarize gene expression in the 8 different networks with the black point designating the mean of log2FC calculated for each condition indicated on the X-axis (factor). The different colors represent the different community clusters (CCs), from CC1 to CC8.

**Supplementary Figure S5. Correlation between the community clusters in the different datasets.** Pearson correlation coefficient was calculated between the different co-expression modules in abiotic (A), biotic (B) and condition-independent (C) datasets. The color indicates the range of PCC with red as a positive PCC and blue as negative PCC values. The higher is the PCC, the higher is the correlation between two given community clusters.

**
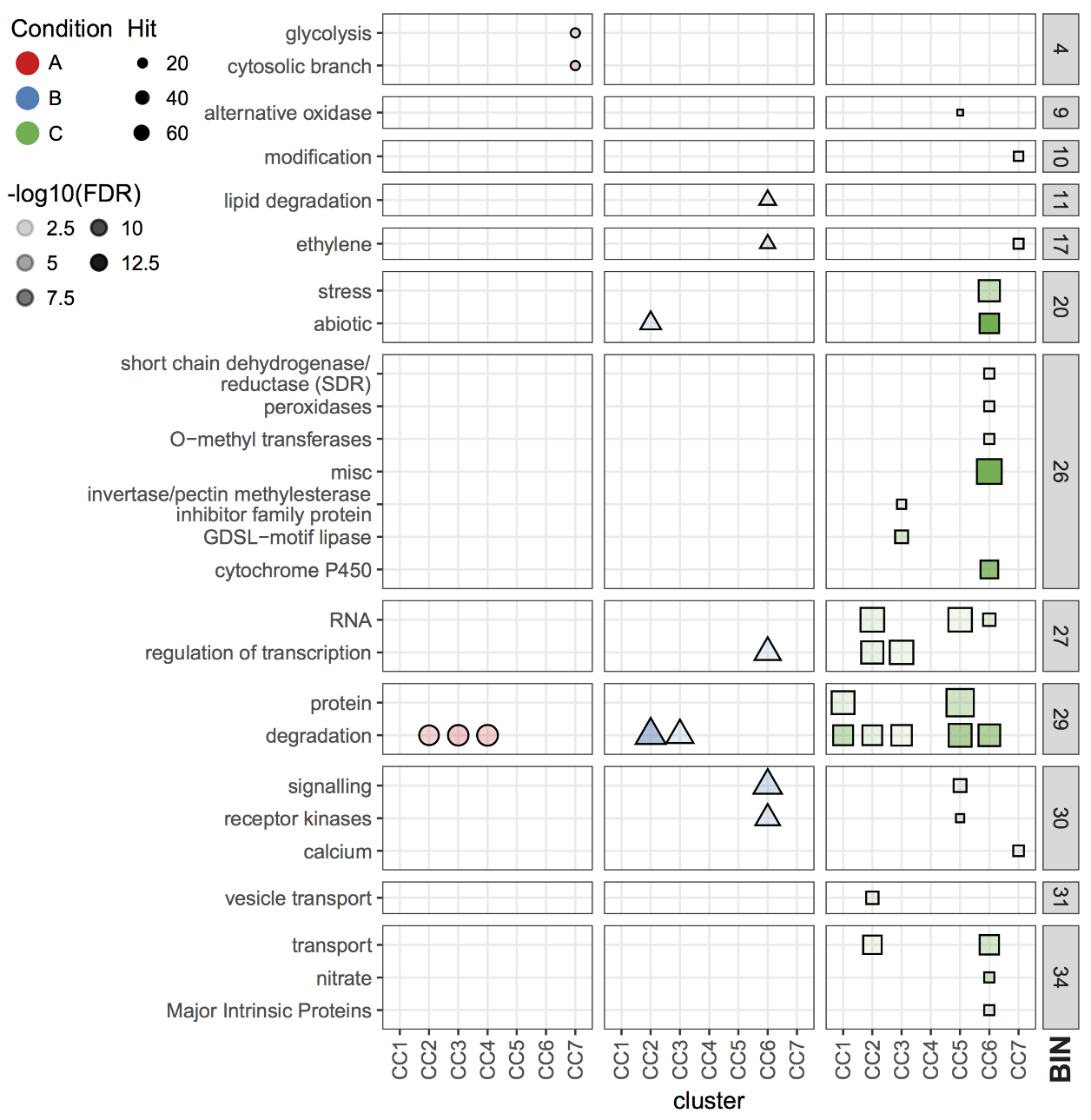
**

**Supplementary Figure S6. Distribution of enriched MapMan BIN categories in each grapevine ATL-centered community cluster under different conditions.** Only the first two levels of the MapMan BIN category hierarchy were considered because they capture the most biological information. The x‑axis indicates the different enriched community clusters divided according to the dataset (A, abiotic, red circles; B, biotic, blue triangles; CI, condition-independent, green squares) and the y‑axis indicates the MapMan BIN descriptions. The size of the symbols (circles, triangles and squares) represents the hit number, i.e. the number of genes belonging to a given MapMan BIN in each community cluster. The level of symbol opacity indicates the FDR/2.5 ranging from 1 to 5. CC, community cluster.


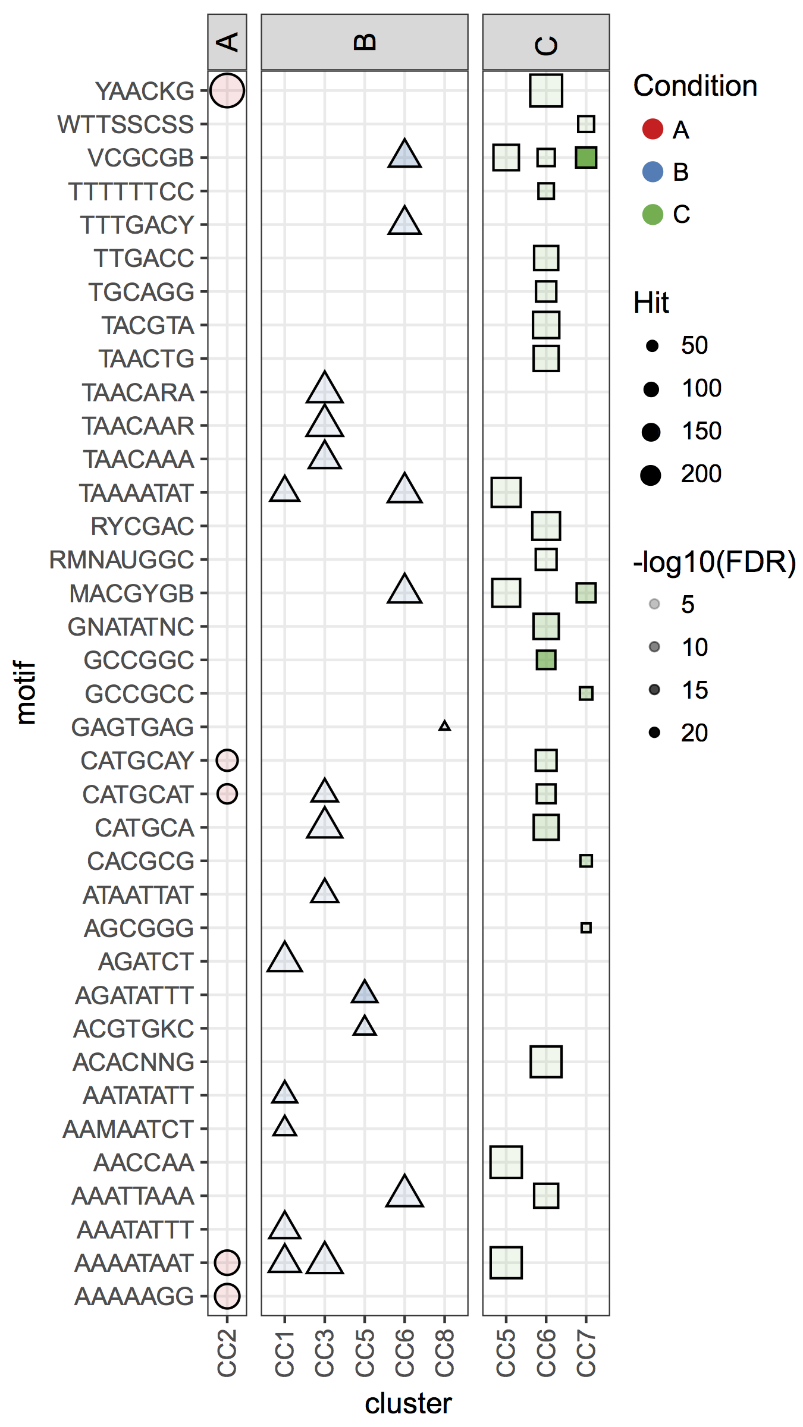


**Supplementary Figure S7. Enrichment of *cis*-acting regulatory elements (CAREs) in grapevine ATL community clusters under different conditions.** Different CAREs enriched in at least one community cluster (CC) under at least one condition are indicated on the left. A, B and C as well as symbols and colors indicate the abiotic (red balls), biotic (blue triangles) and condition-independent (green squares) datasets, respectively. Symbol size represents the number of genes in each ATL community cluster containing a given CARE (50–200), whereas ball opacity represents the false discovery rate (FDR) expressed as –log10(FDR).


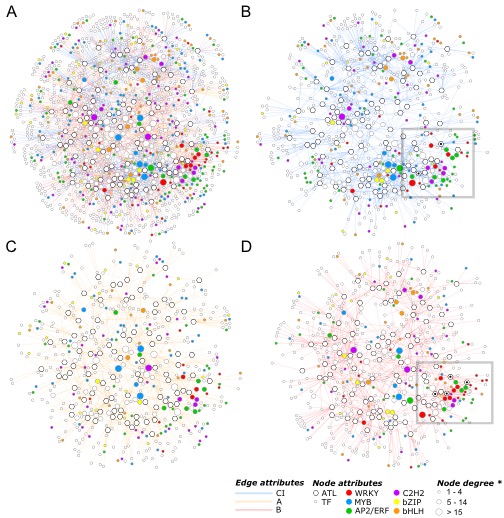


**Supplementary Figure S8**. Combined ATL gene co-expression networks in different conditions highlighting the connection between *ATL*- and transcription factor-encoding genes. Visualization of overlapping (A) or individual condition-independent (B), abiotic (C) and biotic (D) networks. Node colors indicate the dataset (blue, CI; yellow, abiotic; red, biotic). Diamonds represent *ATL* genes, while balls represent transcription factor-encoding genes. Ball colors indicate the TF family as indicated. Diamond and ball size indicates the node degree, i.e. the number of connection for each node, as indicated. CI, condition-independent; A, abiotic; B, biotic; TF, transcription factor.
